# Immunoglobulin G in Platelet-Derived Wound Healing Factors

**DOI:** 10.1155/2021/4762657

**Published:** 2021-01-28

**Authors:** Elisa Seria, Sarah Samut Tagliaferro, Doreen Cutajar, Ruth Galdies, Alex Felice

**Affiliations:** ^1^Department of Physiology and Biochemistry, Faculty of Medicine and Surgery, Centre of Molecular Medicine and Biobanking, University of Malta and Division of Pathology, Mater Dei Hospital, Malta MSD2080; ^2^Department of Surgery, Faculty of Medicine and Surgery, University of Malta Medical School and Mater Dei Hospital, Malta MSD2080

## Abstract

We intended to reformulate an existing platelet-derived wound healing formula to target each phase of the healing wound with the appropriate phase-specific molecules. A decreased perfusion of the skin, often associated with conditions such as thalassemia, sickle cell disease, diabetes mellitus, and chronic vascular disease, is the most common etiology of cutaneous ulcers and chronic wounds. We had previously shown that a PDWHF topically applied to a chronic nonhealing ulcer of a *β*-thalassemia homozygote stimulated and accelerated closure of the wound. The PDWHF was prepared from a pooled platelet concentrate of a matching blood group, consisting of a combination of platelet *α*-granule-derived factors. Processing of the apheresis-pooled platelets yielded various amounts of proteins (3.36 g/mL ± 0.25 (SD) (*N* = 10)) by the better lysis buffer method. Immunoglobulin G was found to be the most abundant *α*-granule-secreted protein. Equally broad quantities of the IgG (10.76 ± 12.66% (SD) (*N* = 10)) and IgG/albumin ratios (0.6 ± 0.4 (SD) (*N* = 10)) were quantified. We have developed a method using a reformulated lysis buffer followed by size exclusion chromatography and affinity chromatography to extract, identify, quantify, and purify IgG from activated platelets. IgG purification was confirmed by Western blot and flow cytometry. It was thought unlikely that the platelet IgG could be accounted for by adsorption of plasma protein, though the variable quantities could account for diversity in wound healing rates. The IgG could protect the wound even from subclinical infections and functionally advance healing. It may be useful in the management of skin ulcers in the early phase of wound healing.

## 1. Introduction

Cutaneous wounds are seldomly caused by decreased perfusion of the skin that becomes infected. Clinical hemolytic disorders such as sickle cell disease (SCD) and beta-thalassemia (*β*-thalassemia) are associated with indolent leg ulcers, partly caused by peripheral hypoxia [1, 2], but may develop further as a result of low bioavailability of nitric oxide (NO), iron overload, and impaired endothelial function [3]. Hemoglobin disorders are frequently observed in Malta, Southern Europe, and the Mediterranean due to several *β* globin gene mutations [4].

Chronic leg ulcers may also develop in individuals with type 2 diabetes mellitus (T2DM) and long-term venous insufficiency. Irrespective of the different pathophysiology, these ulcers indicate poor skin perfusion, altered metabolism, and chronic inflammation, which impairs repair and closure of the wound [5–7].

Ulcer formation results in the release of danger signals, such as pathogen-associated molecular patterns (PAMPs) and danger-associated molecular patterns (DAMPs), as well as other proinflammatory cytokines, which activate the early inflammatory phase of the wound. Subsequently, neutrophil apoptosis triggers macrophages to transition to an anti-inflammatory cellular phenotype. In combination with other local immune signals, this leads to the fundamental steps for the resolution of the inflammation [8]. Iron overload, hypoxia, and hyperglycemia inhibit this physiological transition. The persistent inflammation, possibly augmented by subclinical infection, suppresses the healing of the wound. The oxidative injury may further give rise to the apoptosis of cells in the surrounding tissue [9, 10]. A major regulatory role of the wound repair process is played by platelets that activate signaling stimuli for all the cells involved [11].

There are several possibilities for improvement of the current treatment of leg ulcers because the underlying condition remains incompletely defined. Surgical hygiene, compression bandaging, and limb elevation have been the most common and effective treatments to date [12]. Neither recombinant platelet-derived growth factor (PDGF) nor hydrogels or hydrocolloids, with alginate or carbon, have resulted in robust, repeatable, and favorable treatment outcomes. The previous success of the use of a mixed platelet-derived wound healing formula (PDWHF) on *β*-thalassemia ulcers [13] showed that the diversity of molecules in platelets supports healing. The long-term aim of this study is to reformulate this PDWHF to target different states in the wound healing process in order to produce the ideal mixture required for effective wound healing.

A novel PDWHF preparation was developed from a pooled platelet concentrate of a matching blood group, using a modified lysis buffer. It consisted of a combination of platelet-secreted growth factors, including platelet-derived growth factor (PDGF), platelet-derived angiogenesis factor (PDAF), transforming growth factor-beta (TGF-*β*), platelet factor IV (PF4), and platelet-derived epidermal growth factor (PDEGF), secreted by the *α*-granules [14]. Since this lysis method induced platelet activation, the preparation is additionally expected to contain immunoglobulin G (IgG) [15, 16].

Immunoglobulins form part of both the innate and adaptive immune systems. IgG, together with T cells, is responsible for protection during the first bacterial, fungal, or viral infection and also provides long-term protection through memory B cells [17].

The interactions between immunoglobulins and immune effector cells, such as dendritic, monocyte, and macrophage cells, as well as various subsets of T and B cells, represent the basis for their therapeutic application in the treatment of infectious diseases [18, 19].

Immunoglobulins play a critical role in the immune response as they specifically recognize and opsonize pathogens, bind and neutralize toxins, or act in type II and type III hypersensitivity responses [20–22]. Immunoglobulins have been increasingly employed in the treatment of a variety of medical conditions, such as autoimmune and inflammatory disorders, and also for a variety of infectious diseases and infection-related disorders [23].

They may function as a passive immunotherapeutic or act through functional mediation through the C-terminus of the molecule. Currently, the two FDA-approved indications for immunoglobulin administration are Kawasaki disease (KD) and pediatric HIV infection to prevent secondary infections. There is evidence of a beneficial effect of IgG administration in the prevention of lung infections in patients with primary or acquired immunodeficiencies [24].

This study proposes the identification and quantification of IgG in the PDWHF. Although the initial objective of this long-term research was to define the composition of the PDWHF and the best reformulation so as to better match the requirements of the wound as it develops and heals, the abundance of IgG in this formula suggests a role perhaps bigger than previously thought. Here, we documented a novel method using a reformulated lysis buffer followed by size exclusion chromatography (SEC) and affinity chromatography to extract, identify, quantify, and purify IgG from activated platelets.

The use of immunoglobulins for therapeutic purposes in wound healing is a potential advancement in clinical care. Therapeutic antibodies present numerous opportunities for the treatment of chronic nonhealing wounds [25].

## 2. Materials and Methods

This research was approved by the University of Malta Research Ethics Committee with protocol reference number 56120L7. Apheresis-pooled, leukocyte-depleted platelet products were used as a source for the platelet extracts courtesy of the National Blood Transfusion Center.

### 2.1. Extraction of Proteins

Two methods were evaluated in order to obtain the proteins from the PDWHF: the calcium chloride method [26] and the lysis buffer method [27] with a modification in the lysis buffer composition consisting of 0.9% sodium chloride (NaCl), 0.3% ammonium phosphate ((NH_4_)_3_PO_4_), and 0.3% sodium dihydrogen phosphate (NaH_2_PO_4_) (Sigma-Aldrich, Munich, Germany). The resulting PDWHF extracts were filtered through 0.22 *μ*M MF-Millipore MCE membranes (Merck, Munich, Germany), quantified by the Bio-Rad Bradford protein assay (Bio-Rad Laboratories, California, USA), and then analyzed by High-Performance Liquid Chromatography (HPLC). Protein assay quantification was done in triplicate for each of the 10 PDWHF samples treated with both methods, and the mean ± standard deviation (SD) was calculated using IBM SPSS Statistics.

### 2.2. Flow Cytometry Analysis of Platelet-Associated IgG

Immunofluorimetric analysis of platelet-associated IgG was performed on resting and activated platelets using anti-CD62p-FITC (P-selectin clone AK4, BioLegend, Amsterdam, the Netherlands) as a marker of platelet activation and alpha-granule release [28] and anti-human IgG (clone HP6017, BioLegend) for IgG detection. PE-IgG1 and FITC-IgG1 (clone MOPC-173 and clone MOPC-21, BioLegend) were used as corresponding isotype controls. For immunophenotyping of the resting and activated platelets, the apheresis donor platelet concentrates were centrifuged for 20 min at 300 × g at 4°C. The platelet pellet was resuspended in 2 mL of PBS for further surface and intracellular staining. Surface staining was done by fixing the cell suspension with 1% paraformaldehyde solution at 4°C for 2 hours to inhibit spontaneous platelet activation and then stained with 100 *μ*L of each moAb solution for 20 min in the dark at room temperature. Cells were then washed in 1 mL of PBS and resuspended in 200 *μ*L of PBS. Intracellular staining was done according to the protocol of the manufacturer's kit (Cyto-Fast™ Fix/Perm Kit, BioLegend). For the immunophenotyping of the activated platelets, the platelet pellet was treated with a newly modified lysis buffer to activate the platelets [27]. The activated platelet suspension was stained with 100 *μ*L solution of each moAb for 20 min in the dark at room temperature and subsequently treated with 1 mL of cold 1% paraformaldehyde solution at 4°C in the dark for 20 min prior to data acquisition. All flow cytometry analysis was performed using a FACSCalibur Flow Cytometer (Becton Dickinson, Oxford, UK), and the data was analyzed using the CellQuest software.

### 2.3. Protein Separation and Isolation

HPLC was conducted on a Shimadzu model system 0180 chromatography system (Shimadzu Corporation, Japan). Proteins were separated from 10 samples on a Bio-Sil SEC250 Size Exclusion Column (Bio-Rad Laboratories) and on a hydroxyapatite (HPHT) affinity column (Bio-Rad Laboratories) for selective IgG isolation. Eight samples were analyzed separately on both columns. Two additional samples were first analyzed on the SEC chromatographic column, and the eluant product was subsequently analyzed using the HPHT column. SEC (molecular sieve) chromatography conditions are as follows: isocratic solution: 0.1 M trisodium phosphate (Na_3_PO_4_) and 0.15 M NaCl; pH: 6.57; temperature: 28°C; flow rate: 0.8 mL/min; injection volume: 50 *μ*L; and wavelength: 280 nm. Affinity chromatography conditions are as follows: isocratic solution: 10 mM Na_3_PO_4_ and 0.01 mM calcium chloride (CaCl_2_); pH: 6.8; temperature: 28°C; flow rate: 0.8 mL/min; injection volume: 20 *μ*L; and wavelength: 280 nm. The protein standard mixture (Sigma-Aldrich) was used as a reference for the identification of proteins according to their molecular weight. The native human IgG standard (Abcam, Massachusetts, USA) served as a reference for IgG identification. Bovine serum albumin (BSA) (Sigma-Aldrich) was used as a reference for albumin identification. All three standards were used at a concentration of 10 mg/mL. The harvested IgG protein was dialyzed using Zeba Spin Desalting Columns, 40 K MWCO, 5 mL (Thermo Fisher Scientific, Massachusetts, USA), and concentrated with Pierce Protein Concentrators, 100 K (Thermo Fisher Scientific).

### 2.4. Protein Identification: Isoelectric Focusing (IEF)

IgG was identified according to its isoelectric point (pI) by IEF on agarose gel using the RESOLVE Hemoglobin Kit (PerkinElmer, Massachusetts, USA), which contained ampholytes at pH 6-8. Hemoglobin (Hb) was used as an internal control, and the human IgG standard (Abcam) was used as a reference for IgG identification. The human IgG standard and the harvested IgG from the HPHT column were loaded at a concentration of 10 *μ*g/*μ*L. IEF parameters are as follows: watts: 20 W; volts: 1500 V; and time: 55 min. Focused IgG protein was fixed on the gel with 10% trichloroacetic acid (TCA) for 10 min using the rocking platform. Following fixation, the gel was soaked in deionized water for 30 min. The water was changed three times in the process. The gel was dried at room temperature overnight, after which it was ready for observation.

### 2.5. Protein Identification: WB

The IgG standard and the harvested IgG from the HPHT column were loaded at a concentration of 10 *μ*g/*μ*L. WB parameters are as follows: volts: 120 V; time: 60 min. The proteins were transferred onto a nitrocellulose membrane at 0.3 A for 120 min and blocked overnight at 4°C on a rocking platform. The purified mouse anti-human IgG primary antibody (clone HP6017, BioLegend, London, UK) was diluted with the proportion of 1 : 1000 in 1x Tris-buffered saline (TBS). The membrane was incubated overnight at 4°C and then washed with 1x Tris-buffered saline with Tween 20 (TBST) and 1x TBS. The goat anti-mouse IgG secondary antibody (polyclonal, LI-COR Biosciences, Bad Homburg, Germany) was added and incubated overnight at 4°C. The secondary antibody was diluted at 1 : 15,000. After the second incubation, the membrane was washed and analyzed on a LI-COR Odyssey Infrared Imaging System (LI-COR Biosciences).

### 2.6. Protein Identification: FCM

The harvested IgG was bound to Protein A beads (Bangs Laboratories, Indiana, USA) for FCM assay and analyzed on a FACSCalibur Flow Cytometer (Becton Dickinson, New Jersey, USA) according to the manufacturer's protocol. The human IgG standard and the harvested IgG from the HPHT column were used at 10 *μ*g/*μ*L. The immunocomplex was labeled with 10 *μ*L Phycoerythrin- (PE-) anti-human IgG (clone HP6717, BioLegend). The acquisition was set up at log Forward Scatter (FSC)/Sideward Scatter (SSC) and for PE at fluorescence 2 (FL2) with excitation at 565 nm and emission at 400 nm. Readings were done in triplicate. All the acquisitions and data analyses were performed using the CellQuest software (Becton Dickinson).

## 3. Results

### 3.1. Flow Cytometry Analysis of Platelets

**Figure 1:**
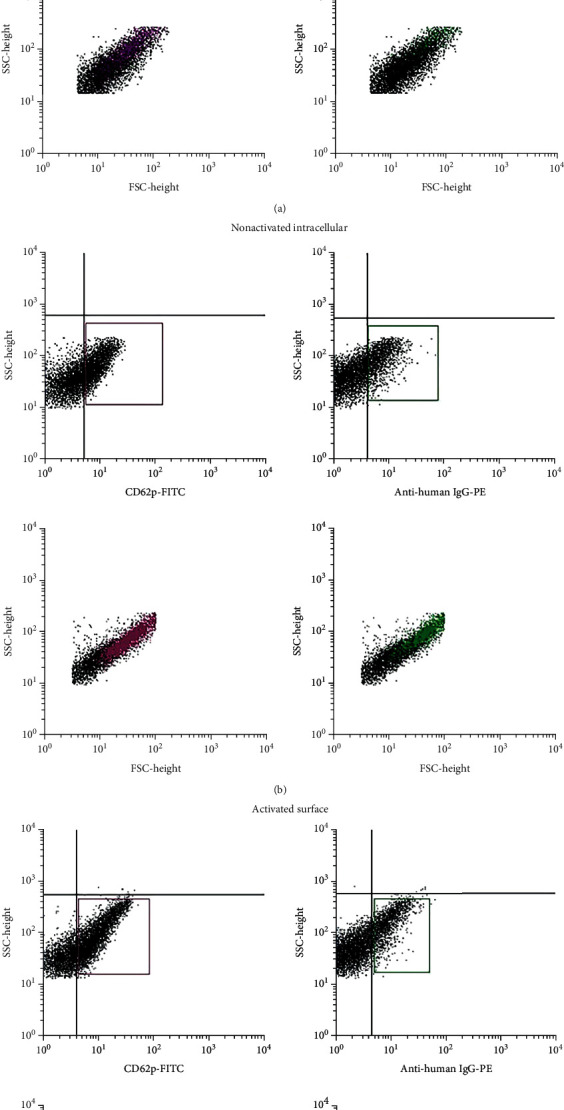
Flow cytometry analysis of platelet-associated IgG. Analysis conducted on nonactivated platelets (surface (a) and intracellular staining (b)) and activated platelets (c) showed different expression levels of the CD62p and IgG. A higher expression was noted in the intracellular staining of nonactivated platelets and in activated platelets when compared with the surface staining of nonactivated platelets.

FITC- and PE-conjugated isotypes were used as a reference for the absence of fluorescence (<10^1^ log scale). An absence of CD62p and IgG was noted on the plasma membrane surface of nonactivated platelets (12.7 ± 6.08% (SD) (*N* = 10) and 3.2 ± 2.5% (SD) (*N* = 10), respectively, of the R2 (gated) population) (Figure 1(a)). On the other hand, the presence of CD62p and IgG was confirmed within the alpha-granules of nonactivated platelets (42.03 ± 3.08% (SD) (*N* = 10) and 26.74 ± 2.88% (SD) (*N* = 10), respectively, of the R2 (gated) population) (Figure 1(b)). An increase in the expression of CD62p and IgG occurred upon platelet activation as a consequence of the degranulation of alpha-granules (from 12.7 ± 6.08% to 57.50 ± 2.70% and from 3.2 ± 2.5% to 27.56 ± 24.09%, respectively) (Figure 1(c)). The IgG expression level upon activation was similar to that found within the alpha-granules before activation. This supports our hypothesis that IgG is released into the surrounding environment upon the degranulation of alpha-granules.

### 3.2. Platelet Protein Extraction

**Figure 2:**
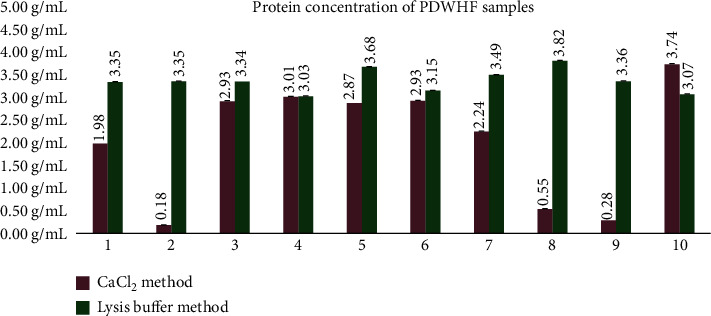
Protein concentrations of the 10 PDWHF extract samples obtained with both the lysis buffer and calcium chloride methods. The concentration range for the 10 samples treated with the calcium chloride method was between 0.18 and 3.74 g/mL with an SD between 0.001 and 0.10 g/mL. The concentration range for the 10 samples treated with the lysis buffer method was between 3.03 and 3.82 g/mL with an SD between 0.001 and 0.003 g/mL. The average concentration for the 10 samples treated with the lysis buffer method was 3.36 ± 0.25 g/mL (SD) (*N* = 10), while the average concentration for the 10 samples treated with the calcium chloride method was 2.07 ± 1.29 g/mL (SD) (*N* = 10).

The concentration range for the samples treated with the calcium chloride method was between 0.18 and 3.74 g/mL with an SD between 0.001 and 0.10 g/mL (Figure 2). The concentration range for samples treated with the lysis buffer method was between 3.03 and 3.82 g/mL with an SD between 0.001 and 0.003 g/mL (Figure 2). The yield of the lysis buffer method was more abundant (3.36 ± 0.25 g/mL (SD) (*N* = 10)) than that of the calcium chloride method (2.07 ± 1.29 g/mL (SD) (*N* = 10)), with better consistency (CoV 0.07 vs. 0.62).

### 3.3. Protein Separation and Isolation: SEC

**Figure 3:**
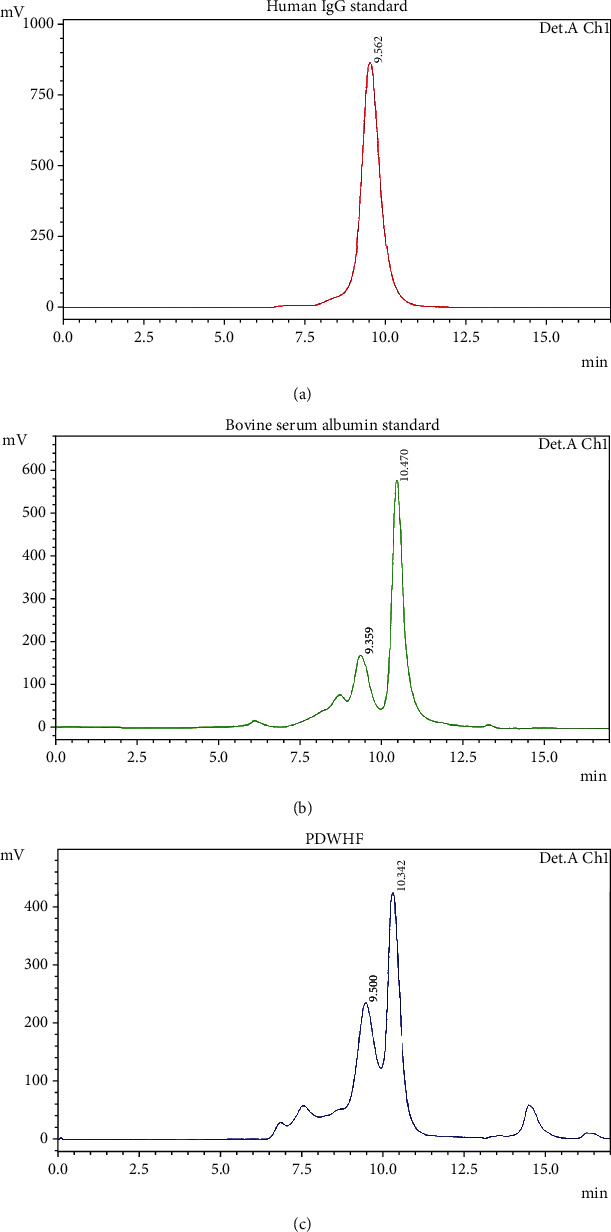
SEC chromatograms. (a) Human IgG standard. Chromatograms obtained with SEC using the Bio-Sil SEC250 Column showed a single zone at elution time 9.5 min for the human IgG standard. (b) Bovine serum albumin standard. Chromatograms obtained with SEC using the Bio-Sil SEC250 Column showed that the bovine serum albumin standard was eluted at two zones with elution times of 9.35 and 10.47 min. The albumin standard contained ≤0.05% IgG, which can correspond to the zone at 9.35. (c) Typical PDWHF. Chromatograms obtained with SEC using the Bio-Sil SEC250 Column showed more zone peaks at elution times from 5 min to 20 min for the PDWHF. The zone eluting at 9.5 min coincided with the elution time of the IgG standard, and the zone that was eluted at 10.3 min corresponded to the elution time of the serum albumin standard.

The separation of the protein standard mixture produced 5 zones at the appropriate time according to the reference values of the manufacturer: thyroglobulin MW 670 kDa at 7 min, *γ*-globulins bovine MW 150 kDa at 9 min, albumin chicken MW 44.3 kDa at 11 min, ribonuclease A type I-bovine MW 13.7 kDA at 11.8 min, and p-aminobenzoic acid at 14 min (data not shown). The IgG standard was eluted at one zone (Figure 3(a)) with an elution time of 9.5 min. The albumin standard was eluted at two zones, 9.35 and 10.47 min (Figure 3(b)). The standard contained ≤0.05% IgG, which corresponded to the zone at 9.35 min (Figure 3(b)) [29]. The chromatographic separation of the 10 PDWHF samples on the SEC250 Column showed the presence of 9 to 14 zones eluting between 5 and 20 min (a typical chromatogram is shown in Figure 3(c)). Two major zones eluting at 9.5 and 10.3 min were noted. The zone eluting at 9.5 min coincided with the elution time of the IgG standard and amounted to 10.76 ± 12.66% (SD) (*N* = 10). The second zone that was eluted at 10.33 min corresponded to the elution of serum albumin and amounted to 23.67 ± 18% (SD) (*N* = 10). The IgG/albumin ratio in the PDWHF was calculated to be 0.6 ± 0.4, with a wide C.V. of 0.1-1.1 (Table 1). All 10 PDWHF samples separated using the SEC column showed chromatographs with an IgG protein eluting at 9.45 ± 0.08 min (SD) (*N* = 10). The percentage of IgG of the total proteins in PDWHF was 2.39 ± 2.88% (SD) (*N* = 10; Table 1) as calculated with reference to the relative areas of the IgG zones in the standard and test PDWHF.

### 3.4. Protein Separation and Isolation: Affinity Chromatography

**Figure 4:**
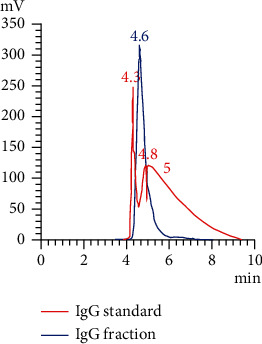
IgG standard zones produced at elution times of 4.3, 4.8, and 5.0 min. The IgG fraction of PDWHF showed a single zone at elution time 4.6 min comparable with the IgG standard chromatogram.

The IgG standard was eluted at different time zones using the HPHT column, relative to the four subtypes as specified by the manufacturer: IgG1: 65%, IgG2: 23%, IgG3: 6%, and IgG4: 6% (Figure 4). All 10 PDWHF extract samples used in this study were separated using the HPHT column and presented with a single zone eluting at 4.65 min, which coincided with the elution time of the IgG standard consisting of IgG 1, 2, 3, and 4 (Figure 4). The percentage of IgG of the total proteins in PDWHF was 0.45 ± 0.43% (SD) (*N* = 10; Table 2) as calculated with reference to the relative areas of the IgG zones in the standard and test PDWHF. The IgG fractions of two PDWHF samples that were eluted from the SEC column were collected and further analyzed on the HPHT column (Table 3).

### 3.5. Protein Identification: Isoelectric Focusing

**Figure 5:**
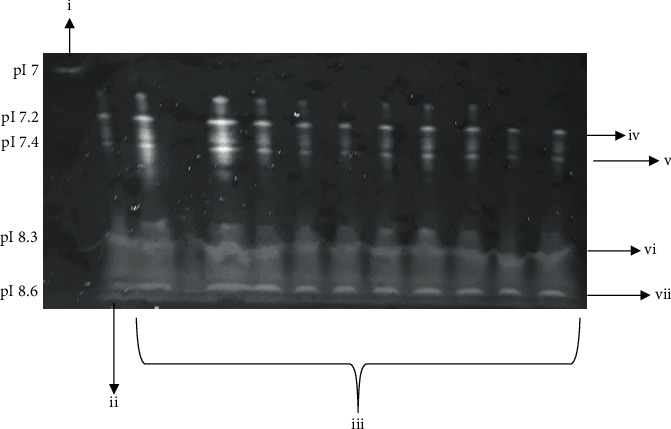
Isoelectric focusing of the 10 IgG harvested from the HPHT column. (i) Hemoglobin reference focused at pI of 7. (ii) IgG standard focused at different pI according to the presence of the four subtypes of IgG. (iii) Harvested IgG focused at different pI according to the presence of the four subtypes of IgG. (iv) IgG4: pI 7.2 ± 0.7. (v) IgG2: pI 7.4 ± 0.6. (vi) IgG3: 8.3 ± 0.7. (vii) IgG1: pI 8.6 ± 0.4.

Hemoglobin was used as an internal control at pH 7 (Figure 5). The IgG reference showed different isoelectric points (pI) between 7.2 ± 0.7 and 8.6 ± 0.4, which corresponded to the four subtypes of IgG (IgG1: 65% pI 8.6 ± 0.4, IgG2: 23% pI 7.4 ± 0.6, IgG3: 6% 8.3 ± 0.7, and IgG4: 6% pI 7.2 ± 0.7) (Figure 5). The 10 harvested IgG from the HPHT column have isoelectric points between 7.2 ± 0.7 and 8.6 ± 0.4, similar to the IgG standard.

### 3.6. Protein Identification: WB

**Figure 6:**
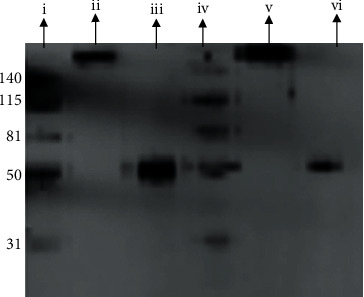
Picture of Western blot. (i) Marker with 140 kDa, 115 kDa, 81 kDa, and 31 kDa bands. (ii) IgG standard not reduced, molecular weight 150 kDa. (iii) IgG standard reduced, molecular weight 50 kDa heavy chain. (iv) Marker with 140 kDa, 115 kDa, 81 kDa, and 31 kDa bands. (v) Harvested IgG not reduced, molecular weight 150 kDa. (vi) Harvested IgG reduced, molecular weight 50 kDa heavy chain.

Both the harvested IgG from PDWHF and the IgG standard showed the same molecular weight of 150 kDa (Figure 6). The gel also showed both the heavy chains at 50 kDa of the collected IgG and the IgG standard when compared with the reference ladder (Figure 6).

### 3.7. Protein Identification: FCM

**Figure 7:**
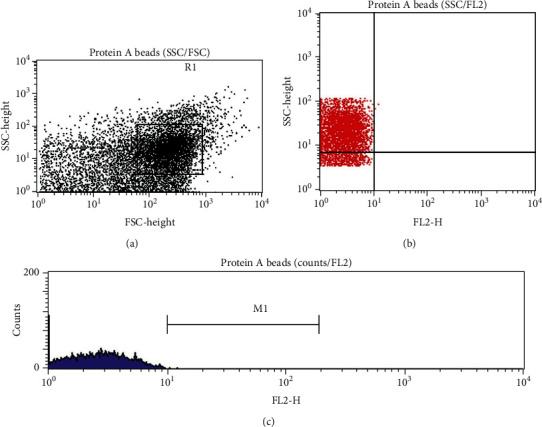
(a) SSC versus FSC dot plot of the unlabeled Protein A beads showing the distribution of the beads. This parameter was used as a reference for the distribution of the immunocomplexes. (b) SSC versus FL2 dot plot of the unlabeled Protein A beads showed no fluorescence (<10^1^ log scale). (c) Histogram counts/FL2 of the unlabeled Protein A beads showed no fluorescence (<10^1^ log scale).

**Figure 8:**
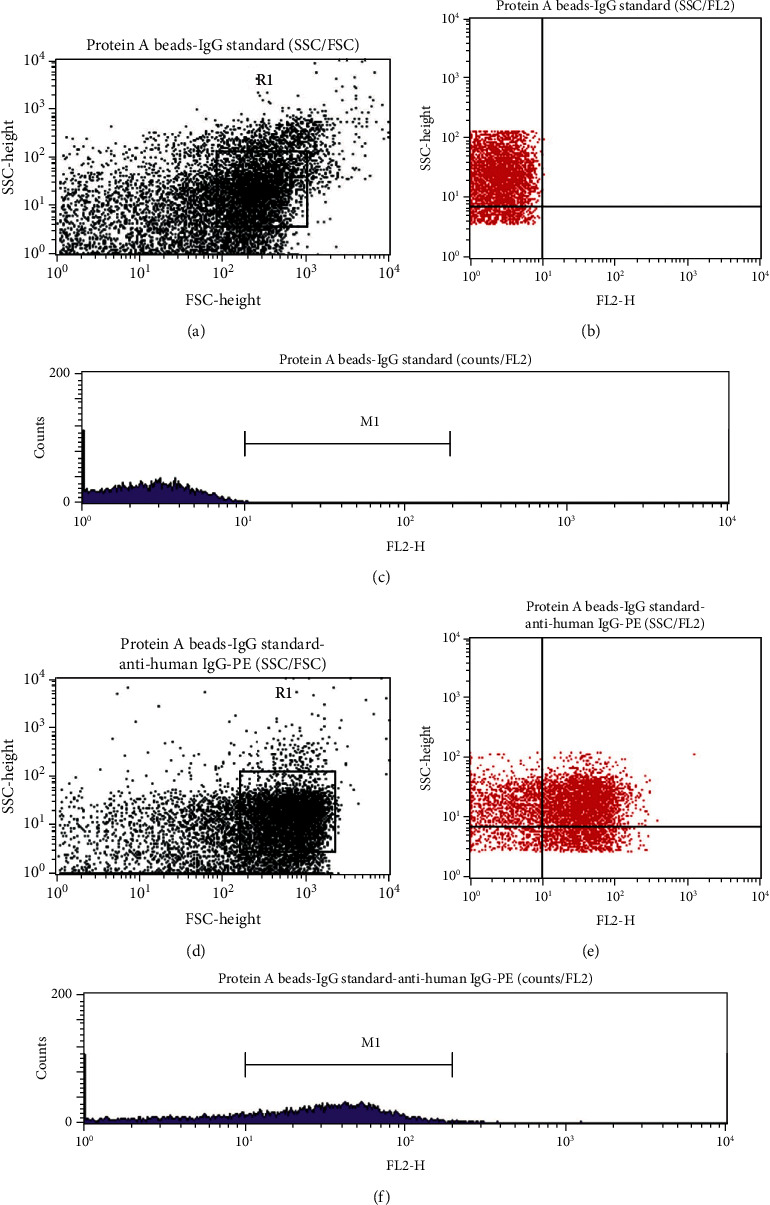
(a, d) SSC versus FSC dot plot of the immunocomplex Protein A beads-IgG standard showed a similar distribution of the unlabeled Protein A beads. (b) SSC versus FL2 dot plot of the unlabeled immunocomplex Protein A beads-IgG standard showed no fluorescence (<10^1^ log scale) in the *x*-axis set up for the FL2-PE channel. (c) Histogram counts/FL2 of the immunocomplex Protein A beads-IgG standard showed no fluorescence (<10^1^ log scale) in the *x*-axis set up for the FL2-PE channel. (e) SSC versus FL2 dot plot of the labeled immunocomplex Protein A beads-IgG standard-anti-human IgG-PE-conjugated showed the presence of fluorescence (>10^1^ log scale) in the *x*-axis set up for the FL2-PE channel. (f) Histogram counts/FL2 of the labeled immunocomplex Protein A beads-IgG standard-anti-human IgG-PE-conjugated showed the presence of fluorescence (>10^1^ log scale) in the *x*-axis set up for the FL2-PE channel.

**Figure 9:**
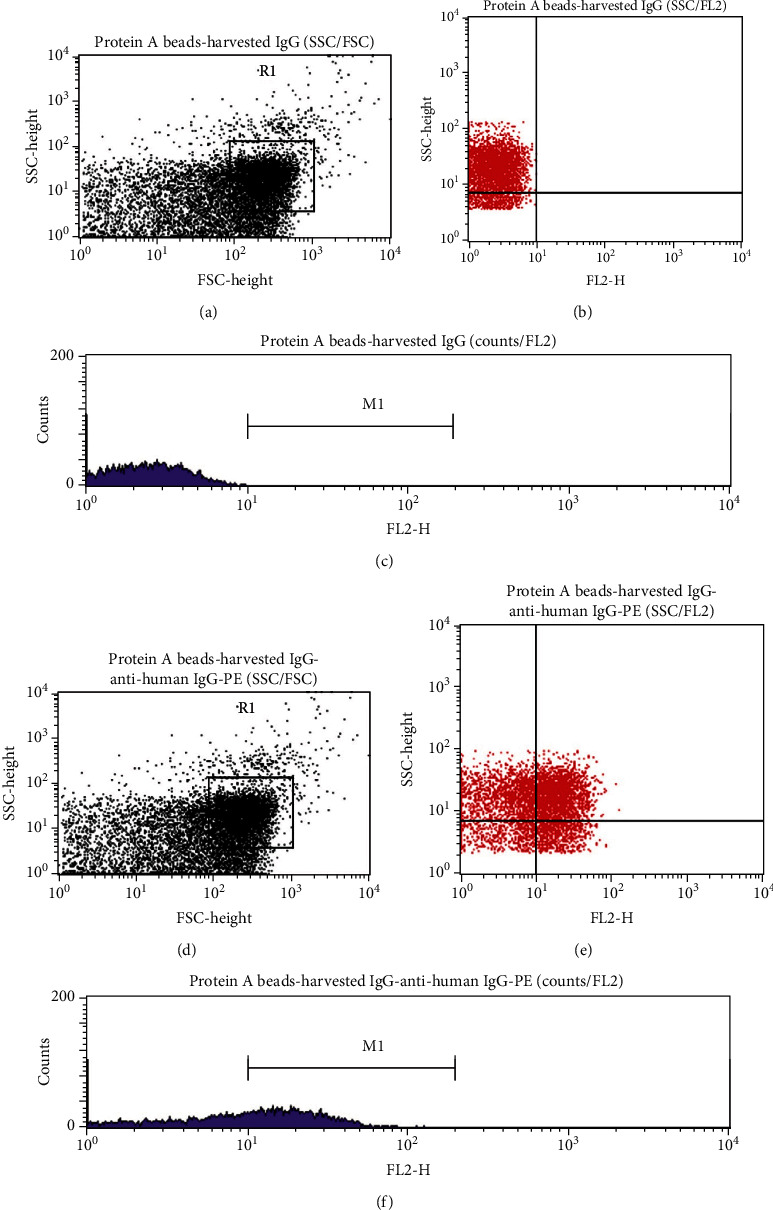
(a, d) SSC versus FSC dot plot of the immunocomplex Protein A beads-harvested IgG showed a similar distribution of the unlabeled Protein A beads. The shift in the FSC parameter is due to the bigger size of the immunocomplex. (b) SSC versus FL2 dot plot of the unlabeled immunocomplex Protein A beads-harvested IgG showed no fluorescence (<10^1^ log scale) in the *x*-axis set up for the FL2-PE channel. (c) Histogram counts/FL2 of the immunocomplex Protein A beads-harvested IgG showed no fluorescence (<10^1^ log scale) in the *x*-axis set up for the FL2-PE channel. (e) SSC versus FL2 dot plot of the labeled immunocomplex Protein A beads-harvested IgG-anti-human IgG-PE-conjugated showed the presence of fluorescence (>10^1^ log scale) in the *x*-axis set up for the FL2-PE channel. (f) Histogram counts/FL2 of the labeled immunocomplex Protein A beads-harvested IgG-anti-human IgG-PE-conjugated showed the presence of fluorescence (>10^1^ log scale) in the *x*-axis set up for the FL2-PE channel.

The unlabeled Protein A beads showed no fluorescence (<10^1^ log scale; Figures 7(b) and 7(c)) The labeled Protein A beads-IgG standard-anti-human IgG-PE-conjugated immunocomplex showed that the IgG standard made up 56.57% of the R1 (gated) population and 33.73% of the entire population (Figures 8(e) and 8(f)). The 10 IgG harvested proteins and their immunocomplexes Protein A beads-anti-human IgG-PE-conjugated made up of 33.4% ± 5.34 (SD) (*N* = 10) of the R1 (gated) population (Figures 9(e) and 9(f)).

## 4. Discussion

In this study, PDWHF from different donors yielded considerable amounts of IgG, even higher than anticipated. We have developed a method using a reformulated lysis buffer followed by size exclusion chromatography (SEC) and affinity chromatography to extract, identify, quantify, and purify IgG from activated platelets. IgG purification was confirmed by subunit analyses on PAGE and immunoprecipitation using flow cytometry.

The platelet products were prepared according to two basic protocols, one designed using plasma to minimize leukocyte and erythrocyte fractions and the other using buffy coats [30, 31]. The two platelet products differed in terms of platelet concentrations and growth factors [32, 33]. The presence of leukocytes may have contributed to the quantity and the types of the released growth factors [34, 35]. Amable et al. showed that alternate separatory procedures influenced the relative composition of the PDWHF [26].

More than 300 proteins can be expected to be identified after thrombin activation of platelets [36]. IgG is one of the most abundant among them [37, 38]. While McMillan et al. suggested that two-thirds of platelet IgG are located on the cell membrane [39], Leporrier et al. found markedly fewer surface IgG [40]. This latter observation was confirmed by George et al., who found that IgG located within platelet *α*-granules and secreted from platelets can present with two known *α*-granular proteins, namely, PF4′ and P-thromboglobulin (BTG) [41].

George et al. showed a good correlation in the total platelet (alpha-granule) content of IgG and plasma. This is shown in a vast range of plasma abnormalities, where the plasma IgG concentration increases in patients with hypergammaglobulinemia but is absent in agammaglobulinemia [42].

In this study, pooled, leukocyte-depleted platelet products were sourced to extract the PDWHF and two different methods of platelet activation were evaluated according to the yield in platelet-stimulated growth factors. Our data showed that the efficiency of the lysis method at yielding growth factors exceeded the calcium chloride method. This may be accounted for by the immediate release of proteins after the disruption of the platelet cell membrane in the lysis method, rather than the slower release of proteins trapped into the fibrin clot in the calcium chloride method. The efficacy of our procedure to activate the platelet was confirmed by the increased expression of the activation marker P-selectin on the cell membrane as a consequence of the degranulation of alpha-granules.

We demonstrated the presence of IgG as an intracellular protein and also noted a higher IgG expression on the membrane of the activated platelets when compared with the expression level on the cell surface of the resting platelets. Our finding suggests that IgG proteins are therefore exposed on the cell membrane after platelet activation and secreted into the surrounding environment. We identified the presence of IgG and serum albumin in varying quantities and proportions in all the 10 PDWHF extracts separated using the SEC column by HPLC. In addition, we determined a selectivity for IgG using the HPHT column.

Other methods for plasma protein fractionation have been used by a number of centers for manufacturing purposes. Most employ a tedious initial five-precipitation step according to Cohn et al. [43], which may follow with the use of one or more chromatographic steps to purify and clean the extracts, with a molecular matrix often being used as a final step, in order to improve albumin purification [44–46]. Although molecular sieves of plasma and PDWHF isolate substantial quantities of IgG and albumin (Figure 1, Table 1), they were nevertheless imbued such that the total recovery after the HPHT affinity chromatography [47–51] was approximately 1%. Modern, high-capacity matrices for chromatography may be suitable to prepare homologous patient-derived molecules for a specific treatment, as in the case of chronic wounds.

As previously developed for the other growth factors [26], further comparative quantification analysis of the IgG content in the PDWHF extracts might be performed through enzyme-linked immunosorbent assay. George et al. investigated and measured the IgG within human platelets by immunocytochemical techniques in response of platelet IgG to agents that cause platelet secretion [41], concluding that IgG is taken up by megakaryocytes and delivered to alpha-granules, where it is stored for later secretion by mature platelets. The Ig/albumin ratio of the PDWHF diverged considerably from that of normal plasma, although the numbers varied considerably between donors.

Patients with leg ulcers are prone to develop an infection, and the influx of IgG triggers the immune system in an attempt to counteract the risk of infection. Antibodies directed to damaged tissue might hence facilitate the removal of bacteria by opsonization, mediated by Fc receptors, which are present on neutrophils and macrophages [52–56]. The Fab region of IgG is hypervariable and provides a wide immune response against many different antigens such as bacterial toxins, while the Fc portion contains sites that are recognized by a class of cellular receptors called “FcgR,” responsible for cell-mediated recognition of antibody-antigen complexes. In our study, we used the properties of the Fc region of the IgG to create an immunocomplex so as to characterize our collected IgG proteins by WB and FCM assays.

Platelet activation leads to the rapid translation of preexisting mRNA, with the release of platelet-secreted proteins, cytokines, exosomes, and microparticles. In fact, upon their activation, platelets release *α*-granular growth factors that are involved in hemostasis, inflammation, antimicrobial defense, immunoregulation, wound healing, and angiogenesis [57].

We used the specific reaction with Protein A to create an immunocomplex between our collected IgG and the Protein A beads. Protein A, which originates from the bacteria *Staphylococcus aureus*, is capable of binding most classes and subclasses of immunoglobulins from various species. Seldomly, Protein A beads are produced using genetically engineered Protein A and are used to purify antibodies, such as by binding with the Fc region of IgG and allowing the Fab region to bind to the antigen [58].

IgG is one of the first proteins present in primary hemostasis, consistent with the role of platelets as inflammatory cells [59, 60]. Nishio and Ito detected immunoglobulin G1 (IgG1) 6-24 h after tissue wounding by using fluorescein isothiocyanate-labeled anti-IgG1 [61].

Extensive research is available on the extraction of platelet-derived growth factors and their application to chronic wounds, but there is scant literature using platelet-associated antibodies. We observed that IgG is a significant component of platelet alpha-granules, and therefore, it plays an important role at the sites where platelets migrate, such as wound sites.

Immunotherapy is regarded as an effective method for the clinical treatment of infectious diseases. More than 60 antibodies are used in a variety of medical fields for the treatments of cancers, autoimmune diseases, and inflammatory disorders [62–64]. Their high binding affinity and their specificity to target proteins are the main characteristics of this success. Recently, immunoglobulins were also shown to be beneficial in many dermatological conditions, such as psoriasis, skin cancer, eczema, and wound healing [65].

A major disadvantage of monoclonal antibody therapy lies in the limited spectrum exhibited by Mab products due to the presence of highly specific antibacterial antibodies targeting one epitope. Polyclonal antibodies exhibit broad specificities against different epitopes or epitope families and therefore have wider applicability in targeted use. Pooled human polyclonal IgG facilitates the elimination of pathogens by stimulating phagocytosis, complement lysis, and oxidative responses by macrophages and neutrophils [53, 55, 66].

Pooled human polyclonal IgG was found to limit the motility of several flagellar pathogens, a factor linked to virulence [67]. Therapeutic antibodies not only neutralize pathogens but also recruit immune cells to engulf the target cell and kill it [68–71]. The topical application of extracted autologous antibodies to the site of a chronic wound would potentially prevent any systemic side effects and also bypass the circulation, thereby presenting a less invasive mode of delivery and a quicker and more efficient mode of action for treatment.

## 5. Conclusion

Very little is known about the role of platelet-associated immunoglobulins in the treatment of chronic wounds. An air-liquid interface skin model will be used to assemble an experimental model of wound healing, simulating the phases and environment of a wound site and investigating the role of the extracted IgG protein. The local application of pooled polyclonal IgG could become a useful therapy to reduce bacterial adhesion and biofilm formation to promote early-stage healing of cutaneous wounds.

## Figures and Tables

**Table 1:** IgG separation from PDWHF samples through size exclusion chromatography.

Sample number	Protein concentration of PDWHF samples measured by Bradford assay (g/mL)	Relative amount of IgG from the total proteins in PDWHF samples (%)	IgG/albumin ratio	% IgG fraction from total proteins in PDWHF
1	3.35	38.57	0.9	8.80
2	3.35	28.71	0.8	6.21
3	3.34	2.66	0.1	0.57
4	3.03	2.96	1.0	0.17
5	3.68	1.64	0.3	0.35
6	3.15	10.95	0.6	1.66
7	3.49	6.04	0.2	1.54
8	3.82	1.12	NA	0.11
9	3.36	7.61	0.1	2.48
10	3.07	7.36	1.1	2.04
Average:	3.36	10.76	0.6	2.39
Standard deviation:	0.25	12.66	0.4	2.88
Range:	3.03-3.82	0.30-38.57	0.1-1.1	0.11-8.80

The table shows the amount of total proteins in the PDWHF measured by Bradford assay, the relative amount of IgG in PDWHF samples measured by the ratio between the IgG area and the total protein area present in PDWHF samples, and the ratio between the IgG and the albumin. The table also shows the percentage of the IgG fraction in PDWHF samples measured by the ratio between the IgG area and the IgG standard area, divided by the total amount of proteins in PDWHF.

**Table 2:** Affinity chromatography.

Sample number	Protein concentration of PDWHF samples measured by Bradford assay (g/mL)	% IgG fraction from total proteins in PDWHF
1	3.35	1.23
2	3.35	1.08
3	3.34	0.15
4	3.03	0.06
5	3.68	0.09
6	3.15	0.31
7	3.49	0.27
8	3.82	0.08
9	3.36	0.70
10	3.07	0.52
Average:	3.36	0.45
Standard deviation:	0.25	0.43
Range:	3.03-3.82	0.06-1.23

The table shows the amount of total proteins in the PDWHF, the IgG fraction present in the HPHT column, and its percentage among the total amount of proteins in PDWHF.

**Table 3:** SEC and affinity chromatography consecutively.

Sample number	Protein concentration of PDWHF samples measured by Bradford assay (g/mL)	% IgG fraction from total proteins in PDWHF	% IgG fraction from total proteins in PDWHF in HPHT after SEC	Ratio area IgG fraction in HPHT/IgG fraction in SEC
1	3.35	8.80	0.052	0.1
2	3.35	6.21	0.036	0.1

The table shows the amount of total proteins in the PDWHF and the IgG fraction recovered in the SEC column in percentage among the total amount of proteins in PDWHF. The table also shows the collected IgG fraction injected on the HPHT column and its percentage among the total amount of proteins in PDWHF. The ratio between the samples' area from the HPHT column and the samples' area from the SEC column has been measured.

## Data Availability

Data are available upon request from the main author.
